# Electroacupuncture at LI11 and SP10 is associated with alleviation of acute urticaria-like reactions in passive cutaneous anaphylaxis: an exploratory analysis of complement-related proteins and multiscale omics

**DOI:** 10.3389/fimmu.2026.1777806

**Published:** 2026-06-08

**Authors:** LinXin You, Yan Zhao, JingYu Zhang, SiJia Li, JiQuan Li

**Affiliations:** 1 Department of Acupuncture and Tuina, Liaoning University of Traditional Chinese Medicine, Shenyang, Liaoning, China; 2 Department of Basic Medicine, Liaoning University of Traditional Chinese Medicine, Shenyang, Liaoning, China

**Keywords:** amino acid metabolism, complement system, electroacupuncture, lipid metabolism, urticaria

## Abstract

**Background:**

The lifetime prevalence of acute urticaria is approximately 20%. Although electroacupuncture (EA) has been investigated as an adjunctive treatment for urticaria, the molecular mechanisms underlying these effects remain elusive. Passive cutaneous anaphylaxis (PCA) serves as an experimental model of localized acute IgE-mediated hypersensitivity in the skin, and it recapitulates the key phenotypic characteristics of acute urticaria. In this study, we leverage an ovalbumin (OVA)-induced PCA model to characterize molecular alterations in the skin and serum following EA intervention at LI11 and SP10 using integrative proteomic and metabolomic analyses.

**Methods:**

An acute urticaria-like model was established via OVA-induced PCA. Rats were treated at the relevant acupoints for 5 consecutive days, during which the blue wheal areas and scratching frequencies were recorded. Immunoassays (ELISA and immunohistochemistry) and histological staining (H&E and toluidine blue) were used to assess cutaneous pathological alterations, vascular permeability, and inflammatory status. Proteomic and metabolomic analyses were conducted on skin and serum samples from three experimental groups to identify differentially expressed proteins and metabolites. An integrative analysis of the omics data was then conducted to explore potential molecular indicators associated with this intervention at both the protein and metabolite levels.

**Results:**

In the acute PCA model, the treatment was concurrent with reduced wheal areas, decreased scratching frequencies, and diminished cutaneous vascular permeabilities. These results suggested attenuated acute inflammatory responses. Multi-omics profiling revealed alterations in the lipid and amino acid metabolic pathways linked to this intervention. The western blot and ELISA results indicated that this intervention coincided with the decreased expression of C3a/C5a and their receptors (C3aR/C5aR) in skin tissues, as well as reduced serum levels of inflammatory mediators (IL-6, TNF-α, and 5-HT).

**Conclusions:**

The results of our study indicated that in the OVA-induced PCA model, this intervention was associated with decreased expression of components of the C3a/C5a-C3aR/C5aR axis and alterations in metabolic profiles. These exploratory findings may inform future mechanistic studies of complement and metabolism associations in this acute model.

## 1 Introduction

Urticaria is a common skin disease characterized by wheal formation and pruritus. According to the symptom duration, urticaria is classified as acute (< 6 weeks) or chronic (≥ 6 weeks) (1). Unlike chronic spontaneous urticaria that involves autoimmune mechanisms (2), acute urticaria results from exogenous allergen-specific IgE-mediated Type I hypersensitivity (3). This cascade is initiated when allergens cross-link IgE-FcϵRI complexes on mast cells (MCs), and this triggers degranulation and the release of histamine and other mediators that cause vasodilation, pruritus, and plasma extravasation (4).

The diagnosis of acute urticaria is primarily based on clinical manifestations and serological evidence. The transient nature of this condition precludes the capture of IgE-mediated early dynamic molecular events in clinical samples. The PCA model serves as an experimental paradigm that recapitulates acute IgE-mediated MC degranulation (5, 6). This model is characterized by allergen (e.g., ovalbumin)-induced vascular hyperpermeability and plasma extravasation. The model mimics the key phenotypic characteristics of acute urticaria (7) and provides a controllable experimental system for the investigation of acute allergic reactions.

Acute urticaria is primarily managed using second-generation H1-antihistamines (1); however, approximately 7.8–24% of patients progress to chronic spontaneous urticaria (8, 9). Upon progression to the chronic phase, a subset of patients exhibit refractoriness to standard therapy (10). Recent investigations have suggested that elevated eosinophil cationic protein and D-dimer levels may serve as biomarkers to identify patients with poor responsiveness to antihistamines (11). EA demonstrates immediate symptom control and long-term immunomodulatory effects for the clinical management of chronic spontaneous urticaria (12–14). Nevertheless, the molecular cascades of acute immediate-type hypersensitivity differ fundamentally from those governing chronic adaptive immune dysregulation. Consequently, mechanisms underlying alleviation of acute IgE-mediated reactions cannot be extrapolated from chronic disease paradigms and remain poorly characterized. In this study, we utilize an OVA-induced PCA model and integrative proteomic and metabolomic analyses to characterize molecular alterations following treatment with EA at LI11 and SP10 to identify acute-phase molecular indicators associated with the alleviation of acute inflammation. We also provide preliminary exploratory data regarding molecular changes coinciding with EA at LI11 and SP10 in this acute allergic model.

## 2 Materials and methods

### 2.1 Grouping animals and the OVA-induced acute urticaria-like model establishment

Eight-week-old male Sprague-Dawley rats were purchased from Changsheng Biotechnology (Liaoning, China) and housed at the Animal Center of the Liaoning University of Traditional Chinese Medicine. The animals were maintained under standard laboratory conditions (temperature: 23 ± 2 °C; regular 12-h light-dark cycle; free access to food and water). The study was reviewed and approved by the Experimental Animal Welfare Ethical Review Committee of Liaoning University of Traditional Chinese Medicine (Approval No. 21000042022118, Date: November 11, 2022). One week after acclimatization, three rats were randomly selected for preparation of the anti-ovalbumin (anti-OVA) serum. These rats were immunized via subcutaneous injection of OVA (10 mg/mL, Yuanye Bio, Shanghai, China) into the back and a concurrent intraperitoneal injection of an aluminum hydroxide adjuvant (0.5 mg/mL). This immunization protocol was performed every other day for a total of three injections. Ten days after the final injection, blood samples were collected from the abdominal aorta under isoflurane anesthesia (RWD Life Science, Shenzhen, China). The serum was separated using centrifugation, diluted 10-fold, and stored at −80 °C until use.

The remaining rats were randomly assigned to the following three groups (n = 10 per group): control, model (OVA), and electroacupuncture (EA). Acute IgE-mediated urticaria-like reactions were induced using the PCA model (15, 16). Following isoflurane anesthesia, the dorsal skin was shaved using an electric clipper and depilatory cream. Three intradermal injection sites were marked on the shaved area that were spaced 1 cm apart. Rats in the OVA and EA groups received intradermal injections of an anti-OVA serum (0.1 mL per site), whereas the control rats received 0.1 mL of 0.9% NaCl at the corresponding sites. These resulted in visible circular bulges. After 48 h, the OVA and EA groups received intravenous injections of 1 mL of 1% OVA mixed with 0.5% Evans blue (1:1, v/v) via the tail vein to induce the urticaria-like reaction. Control animals received an equivalent volume of normal saline via the same route. The sensitized skin areas were evaluated 30 min after challenge by an investigator blinded to the group allocations. Successful establishment of the acute urticaria-like model was confirmed by the presence of Evans blue extravasation, increased scratching behaviors, restlessness, and subsequent histological confirmation of inflammatory alterations in skin tissues.

### 2.2 Grouping treatments and sampling

Rats underwent EA treatment after confirming wheal formation and pruritus. Following this, they were anesthetized using isoflurane inhalation and positioned supine on a heating pad to maintain a body temperature of 37 °C. The acupoint nomenclature and locations followed the standardized guidelines published by the China Association of Acupuncture and Moxibustion (17). The EA group rats received bilateral EA stimulation at Quchi (LI11) and Xuehai (SP10) at a depth of 2–3 mm. Electrical stimulation was delivered using Hua Tuo sterile acupuncture needles (0.18 mm × 13 mm; Suzhou Medical Supplies Factory Co., Ltd., Suzhou, China) with a continuous wave of 2 Hz. Treatments were administered once daily for 20 min over 5 consecutive days. Slight trembling of the bilateral limbs during stimulation indicated a satisfactory electric current delivery. The control and OVA group rats were immobilized in an identical posture for 20 min without the acupuncture intervention. As no sham EA or non-acupoint stimulation was included, the observed differences between the OVA and EA groups may reflect non-specific effects of restraint, needling, electrical stimulation, stress, or somatosensory activation rather than acupoint-specific actions at LI11 and SP10.

**Figure 1:**
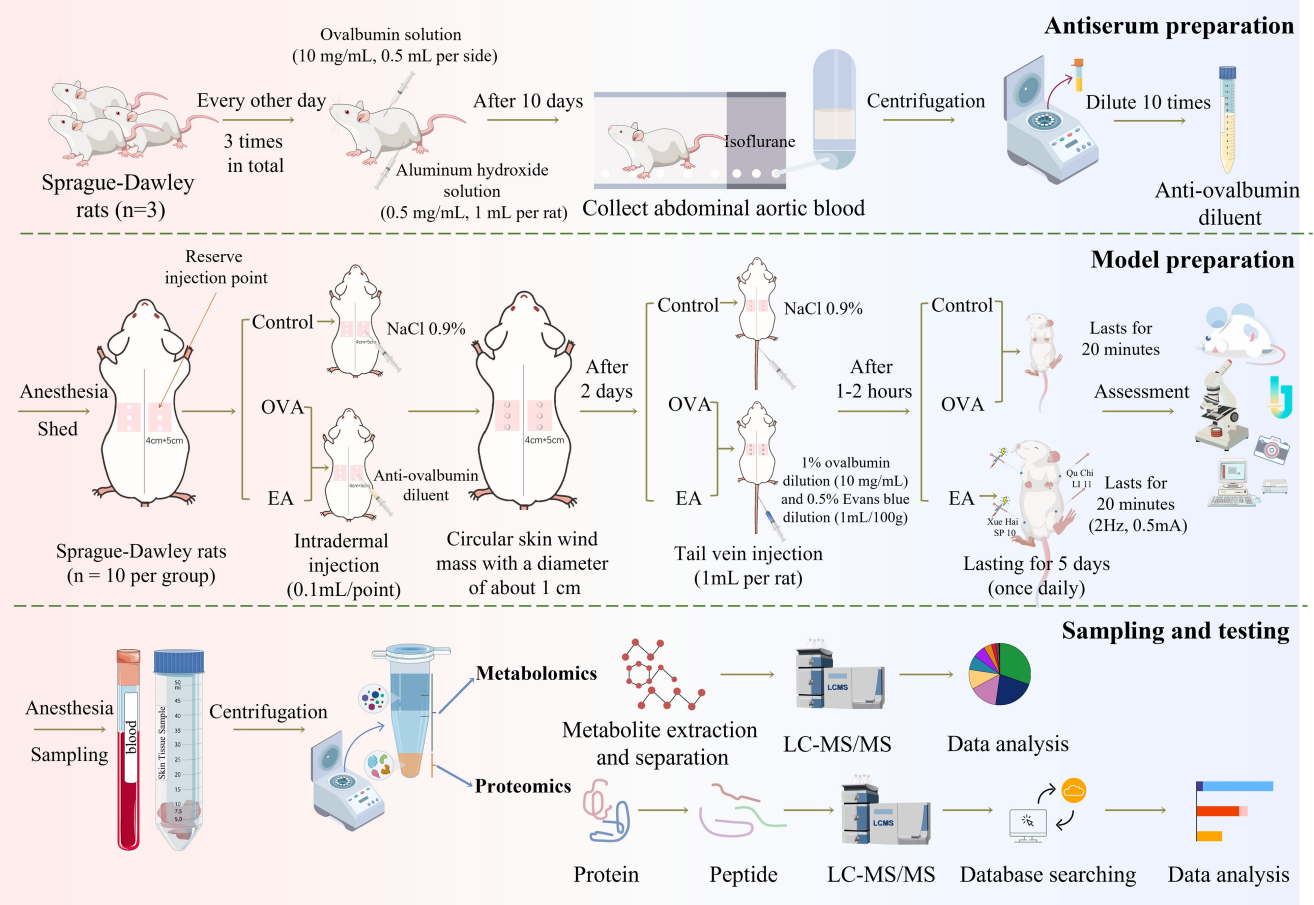
The flowchart of the experiment. n, number of animals; OVA, ovalbumin; EA, electroacupuncture; LC-MS/MS, liquid chromatography-tandem mass spectrometry; Hz, Hertz; mA, milliampere.

Following the final treatment, behavioral assessments (scratching frequencies) were performed on all rats (n = 10 per group). Blood samples were collected from overnight-fasted rats, after which all animals were sacrificed and skin and subcutaneous tissues were harvested from both sides of the back. Blood samples were centrifuged at 7,000 rpm and 4 °C for 15 min (Eppendorf 5430R, Shanghai Pudi Biotechnology Co., Ltd., Shanghai, China). Plasma was collected and stored at −80 °C. Sample allocation was predetermined according to assay requirements. The ELISA and metabolomics analyses utilized plasma from six rats per group. The Evans blue extravasation assays utilized plasma from three rats per group, and these specific plasma samples were excluded from the biochemical analyses due to potential dye interference. Skin samples were processed through three distinct pathways. Samples from three rats per group were fixed in 4% paraformaldehyde for the histopathological analyses (H&E, toluidine blue, and immunohistochemistry). Samples from three rats per group were snap-frozen in liquid nitrogen for the proteomic analysis and western blot assays. Technical triplicates were performed for each biological sample for the western blot assays. Samples from the remaining three rats per group were subjected to Evans blue extravasation area measurements for a vascular permeability assessment. An integrated multi-omics analysis was conducted using matched sample sets from nine rats (n = 3 per group), wherein the skin tissues for the proteomics and plasma for the metabolomics were collected from the same individual rat to enable a paired correlation analysis. The experimental workflow is illustrated in **Figure 1**.

### 2.3 Scratching behaviors and evaluation of the wheal response

The scratching behavior was defined as a rapid, repetitive, and back-and-forth movement of the hindlimb toward the injection site (18). Rats were observed for 30 min using a video recorder, and the frequency of back skin scratching at the sensitization sites was quantified in a blinded manner.

Thirty minutes post-antigen challenge, rats from each group were anesthetized, and photographs of the sensitized regions on their backs were taken. The blue wheal areas were quantified utilizing ImageJ software. Skin tissues from each group of rats were collected for subsequent pathological examinations and proteomic analyses.

### 2.4 Histological analysis

Two independent observers conducted staining on the formalin-fixed, paraffin-embedded skin tissue sections (YD-6L, Jinhua YIDI Medical Appliance Co., Ltd., Jinhua, Zhejiang, China), and both observers were blinded to the samples. The paraffin-embedded sections were cut to thicknesses of 5 μm. This was followed by the removal of paraffin using xylene and rehydration through graded ethanol. The sections were then stained with H&E from YITA Biotechnology Co., Ltd, Beijing, China and toluidine blue (C0052) from Baoman Biotechnology Co., Ltd., Shanghai, China. The histopathological alterations at the sensitization sites on the dorsal skin as well as the connective tissue in the skin of MCs and their degranulation were then examined. The MCs were deemed positive when the cytoplasmic granules exhibited a purple-red stain, and the nuclei exhibited a blue stain. The staining intensities were categorized as follows: 0 (no staining), 1 (weak staining), 2 (strong staining), and 3 (very strong staining). Staining was considered positive when weak-to-very strong staining intensities were observed. The sample slides (×200) were analyzed. The MCs were quantified in three non-overlapping random fields, and the means of both the total and degranulated MCs were calculated. The degranulation rates were calculated as follows: degranulation rate (%) = degranulated MC count/total MC count × 100%. Mast cell degranulation was defined as mast cells with scattered granules or nuclei deformations.

### 2.5 Immunohistochemistry detection

The paraffin sections of the skin underwent antigen retrieval and serum blocking for immunohistochemical staining. They were then incubated overnight at 4 °C with antibodies to the platelet endothelial cell adhesion molecule-1 (PECAM-1 or CD31) (1:200, A01513-3, Boster, Wuhan, China). Incubation with horseradish peroxidase-conjugated secondary antibody was performed at 37 °C for 30 min. Visualizations were achieved using a 3,3’-diaminobenzidine tetrahydrochloride (DAB) chromogenic reaction with hematoxylin counterstaining.

### 2.6 Enzyme-linked immunosorbent assay

The rat serum IgE, HIS, 5-HT, IL-6, and TNF-α levels were measured using ELISA kits (eBioscience, San Diego, CA, USA) according to the manufacturer’s instructions. The optical density (OD) absorbance at 450 nm was assessed using the Synergy™ HTX Multi-Mode Microplate Reader (BioTek).

### 2.7 Proteomics analysis

The proteomic expression profiles of skin tissue samples from the Control, OVA, and EA groups were identified using tandem mass tags (TMTs). Protein samples were prepared using the filter-aided proteome preparation (FASP) method, followed by enzymatic digestion (19) and peptide quantification (OD 280). The peptides were then labeled using the corresponding TMT labeling kit from Thermo. The peptide segments marked in each group were mixed, and the samples were desalted on a C18 Cartridge prior to separation into multiple components using high pH reversed-phase liquid chromatography. Each component was vacuum-dried and stored at –80 °C. A liquid chromatography (LC) with tandem mass spectrometry (LC-MS/MS) analysis was performed on a Q Exactive mass spectrometer in conjunction with an EASY nLC (Thermo Fisher Scientific). In positive ion mode, the mass spectrometer was operated to acquire full MS scans at a resolution of 70,000 at m/z 200 and MS/MS scans at a resolution of 17,500 at m/z 200. The maximum injection time for MS and MS/MS was set to 50 ms. The normalized collision energy was set to 30 eV, and the isolation window was set to 2 m/z. The dynamic exclusion duration was set to 60 s. The raw data files obtained from the mass spectrometer were imported into Mascot 2.2 and Proteome Discoverer 1.4 software for a database search and a quantitative analysis. To annotate the sequences, information was extracted from Blast2GO and KEGG Automatic Annotation Server (KAAS). The Gene Ontology (GO) and Kyoto Encyclopedia of Genes and Genomes (KEGG) enrichment analyses of the identified differentially expressed proteins were performed using Fisher’s exact test, and the interaction networks were constructed to elucidate their biological significance and potential roles under experimental conditions. A clustering heatmap of all proteins was generated using the ComplexHeatmap R package (R Version 3.4). The InterProScan software package was utilized for the domain prediction of differentially expressed proteins. Additionally, the subcellular localization of differentially expressed proteins was determined using an online tool available at http://cello.life.nctu.edu.tw/.

### 2.8 Metabolomics analysis

The serum samples were thawed and stored in Eppendorf tubes at –80 °C, and an appropriate amount of the sample was added to a precooled methanol/acetonitrile/water solution (2:2:1, v/v). The mixture was thoroughly vortexed and subjected to low-temperature ultrasonic extraction for 30 min. The samples were then placed in a –20 °C freezer for 10 min and then centrifuged at 14,000 g for 20 min at 4 °C. After centrifugation, the supernatant was collected and dried under nitrogen, and 100 µL of acetonitrile-water (1:1, v/v) was added to each sample. The samples were vortex mixed for 30 s and then centrifuged at 14,000 g for 15 min at 4 °C. The supernatant was filtered through a 0.22-µm filter membrane and collected into sample vials for the ultra-high-performance liquid chromatography coupled with tandem mass spectrometry (UHPLC-MS/MS) analysis. Equal volume mixing of all samples was performed to prepare the mixed quality control (QC) samples, and a QC sample was inserted every 5–15 samples (20–23). Serum metabolites were analyzed using a liquid chromatography-mass spectrometry (LC-MS) system that consisted of an Agilent 1290 Infinity LC ultra-high-performance liquid chromatography (UHPLC) system coupled with a Triple TOF 6600 mass spectrometer (AB SCIEX). A chromatographic analysis was performed on a Waters ACQUITY UPLC BEH Amide (1.7 µm, 2.1 mm × 100 mm) column. A gradient elution program that utilized the following two mobile phases was employed: A) water + 25 mM ammonium acetate + 25 mM ammonia and B) acetonitrile with a flow rate of 0.35 mL·min^−1^ and a column temperature of 45 °C. The injection volume was 2 mL. An electrospray ionization source (ESI) was used as the ion source. Sample mass spectral signals were collected in both positive and negative ion scanning modes. The mass spectrometry parameters are detailed in **Supplementary Table 1**.

### 2.9 Proteomics and metabolomics joint analysis

Based on the results of the metabolomics and proteomics studies, a joint analysis was conducted. Differential metabolites with variable importance for the projection (VIP) values > 1 and P values < 0.05, as well as differential proteins with P values < 0.05, were selected based on the results of the metabolomics and proteomics differential analysis. We then used the selected differential metabolites and proteins to calculate the correlations and corresponding p-values using the corAndPvalue function from the WGCNA package in R. The correlation results were further filtered, and based on the filtered results, a clustering correlation heatmap was generated using the ComplexHeatmap package in R. Additionally, a correlation chord diagram was plotted using the circlize package in R.

### 2.10 Western blots

Skin tissues were homogenized to extract the total protein. The protein concentration was then measured. Approximately 40 μg of the extract was boiled and separated using sodium dodecyl sulfate–polyacrylamide gel electrophoresis and then transferred to a polyvinylidene fluoride membrane. After blocking with 5% nonfat milk for 1 h at room temperature, these membranes were then each incubated with the corresponding primary antibody overnight at 4 °C. The proteins were detected with anti-C3a (1:500, PK38715; Abmart, Shanghai, China), anti-C5a (1:500, MBS177443; MyBioSource, San Diego, CA, USA), anti-C3aR (1:500, sc-133172) and anti-C5aR (CD88) (1:500, sc-53788; both Santa Cruz Biotechnology, Dallas, TX, USA), and anti-GAPDH (1:1000, #5174; Cell Signaling Technology, Danvers, MA, USA). The membranes were then incubated with a secondary antibody (Thermo Fisher Scientific, Waltham, MA, USA). The protein bands were identified using an electrochemiluminescence ultrasensitive luminescent reagent (Beyotime, Shanghai, China), and their intensities were quantified using Gel-Pro-Analyzer software (Media Cybernetics, USA). Glyceraldehyde-3-phosphate dehydrogenase (GAPDH) was included as a loading control.

### 2.11 Statistical analysis

Data were analyzed using SPSS 26.0 (IBM, NY, USA) and GraphPad Prism 8.0 software. All data are expressed as means ± standard deviations. Comparisons among multiple groups were performed using one-way analyses of variance, while comparisons between two groups were conducted using T-tests. A p-value < 0.05 was considered statistically significant. Statistical significance was defined as *p < 0.05, **p < 0.01, and ***p < 0.0001. Omics screening employed nominal P < 0.05 and VIP > 1 as exploratory selection criteria; no differential features survived stringent multiple testing correction (FDR < 0.05) in the Control vs. OVA comparison.

## 3 Results

### 3.1 EA at LI11 and SP10 coincided with symptoms in the OVA-induced acute urticaria-like model

**Figure 2:**
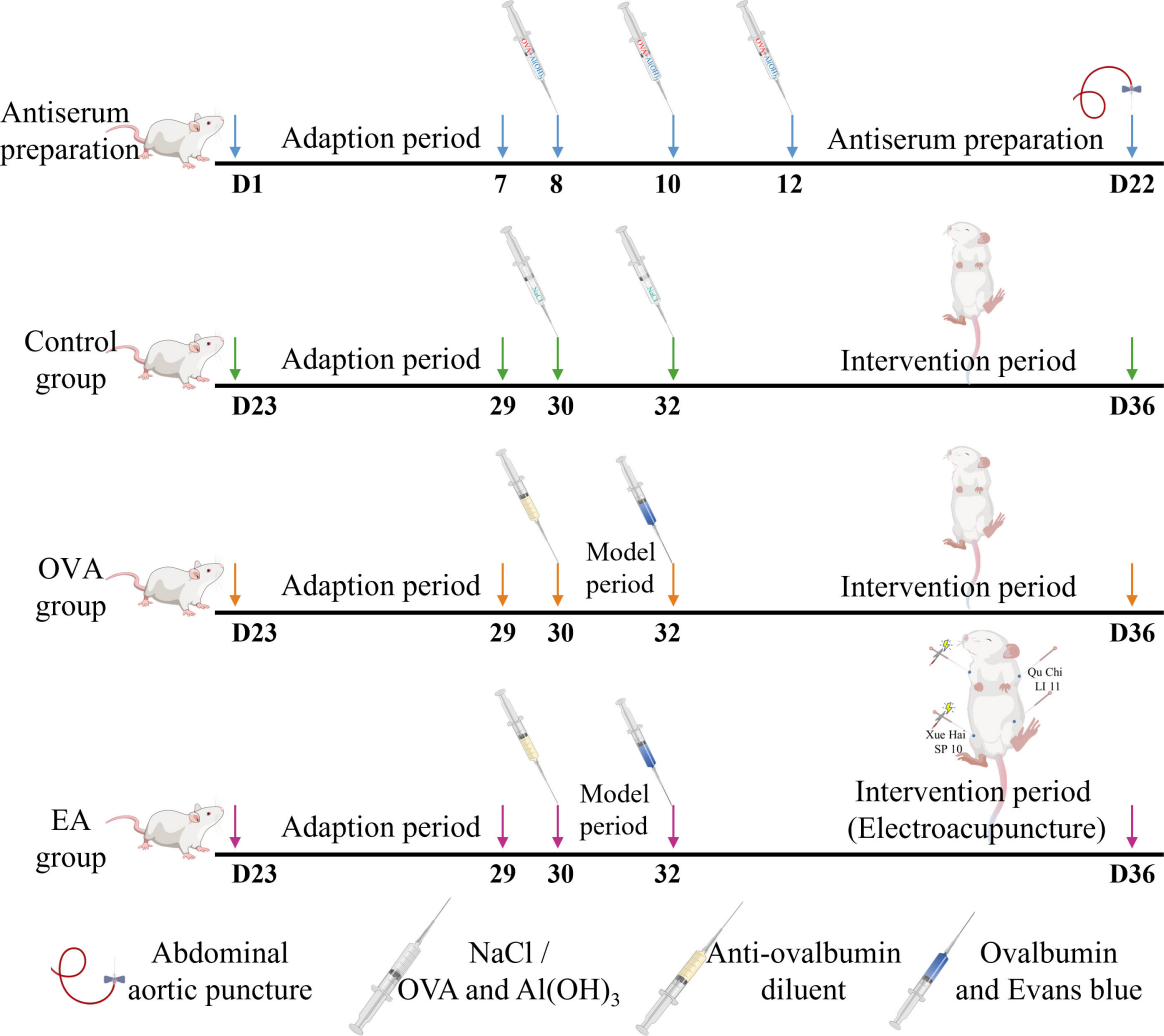
Schematic of the experimental design. D, day; OVA, ovalbumin; EA, electroacupuncture; NaCl, sodium chloride; Al (OH)_3_, aluminum hydroxide.

**Figure 3:**
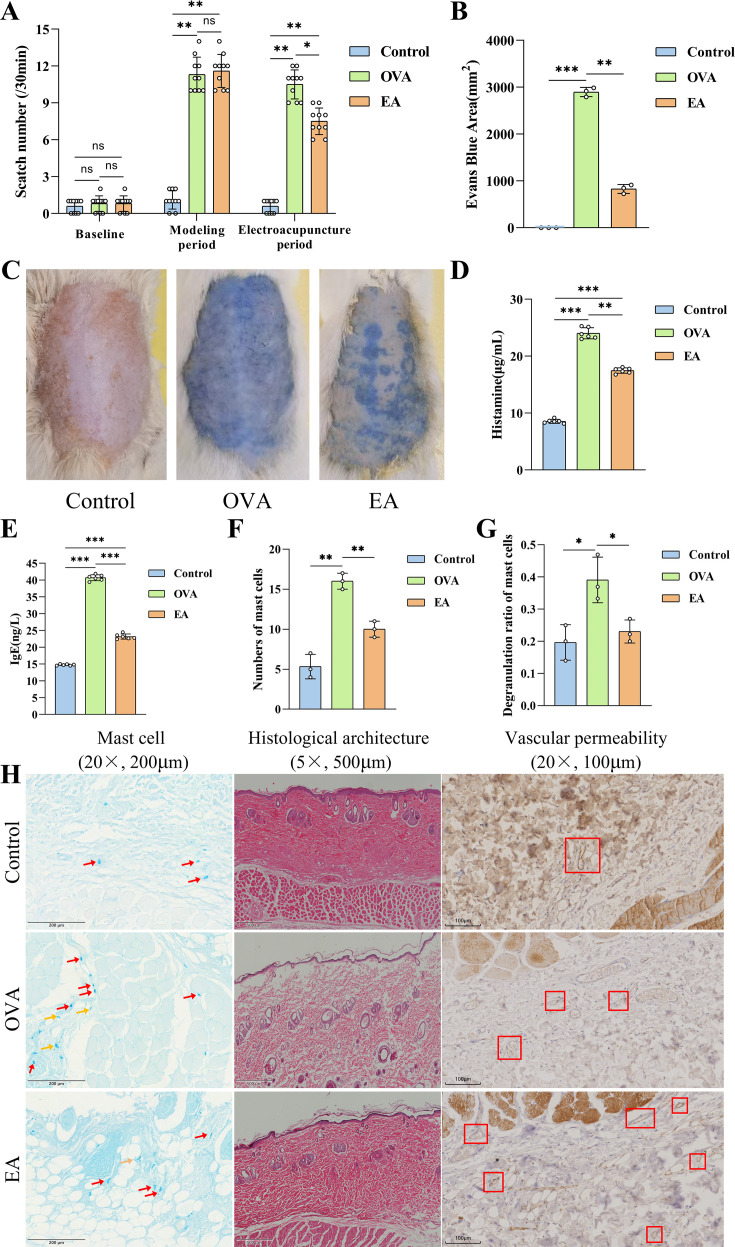
Changes in urticaria-like symptoms and pathological injury following electroacupuncture treatment in a rat model of ovalbumin-induced passive cutaneous anaphylaxis. **(A)** Changes in scratching frequency across different phases (30-min observation windows, n = 10 rats per group). **(B, C)** Evans blue extravasation area quantified by ImageJ software and representative images of the dorsal skin (n = 3 rats per group). **(D, E)** Serum histamine and immunoglobulin E levels measured by enzyme-linked immunosorbent assay (Histamine: μg/mL; (Histamine: μg/mL; immunoglobulin E: ng/L; n = 6 rats per group). **(F, G)** Changes in mast cell counts and degranulation rate after toluidine-blue staining (n = 3 rats per group). **(H)** Representative images showing toluidine blue staining for mast cell identification (n = 3 rats per group), hematoxylin and eosin staining for histological architecture (n = 3 rats per group), and immunohistochemical staining for platelet endothelial cell adhesion molecule-1 to evaluate vascular permeability (n = 3 rats per group). Red arrows indicate non-degranulated mast cells; yellow arrows indicate degranulated mast cells (with scattered granules or nuclei deformation). Red boxes indicate microvascular structures. Data are presented as means ± standard deviations, *p < 0.05, **p < 0.01, ***p < 0.0001, ns, not significant. IgE, immunoglobulin E; Control, control group; OVA, ovalbumin; EA, electroacupuncture.

A rat model of OVA-induced acute urticaria was established to evaluate changes coinciding with this treatment (**Figure 2**). Prior to EA treatment, we verified the existence of blue spots, scratches, restlessness, and pathological inflammatory responses on the skin of UR rats. No differences in scratching behavior were observed among the three groups during the baseline period (P > 0.05). During the modeling period, the number of scratches in the OVA group was significantly higher than that in the Control group, but there was no difference compared to that of the EA group. During the acupuncture period, the number of scratches in the EA group was slightly lower than that in the OVA group, and the result was significantly different compared to that of the Control group (**Figure 3A**). The rats in all groups except for the Control group exhibited conspicuous blue wheals, indicating successful establishment of the UR model (**Figure 3C**). Compared with the OVA group, the EA group exhibited significantly reduced the area of Evans blue (**Figure 3B**) and lower serum levels of histamine and IgE (**Figures 3D, E**). The interaction between leukocytes and the endothelium is mediated by a range of adhesion molecules whose expression is upregulated in response to pro-inflammatory cytokines (24). The platelet endothelial cell adhesion molecule-1 (PECAM-1 or CD31) is predominantly expressed at endothelial cell junctions and is involved in the diapedesis of leukocytes (25). Additionally, it modulates immune responses and vascular integrity (24, 26). Toluidine blue staining of the back skin sections revealed a reduction in degranulated mast cells (MCs) following treatment. H&E staining and PECAM-1 immunohistochemistry revealed that, following the EA intervention, the collagen fiber gaps were only mildly widened and showed no obvious decoloration. This was accompanied by a marked increase in microvessel formation. Typical UR-associated histopathological alterations, including edema, capillary dilatation, and inflammatory cell infiltration, were all alleviated to varying extents (**Figures 3F–H**). These results suggested that symptom alleviation was observed following EA at LI11 and SP10 in the OVA-induced acute urticaria-like model.

### 3.2 Identification of differentially expressed proteins

**Figure 4:**
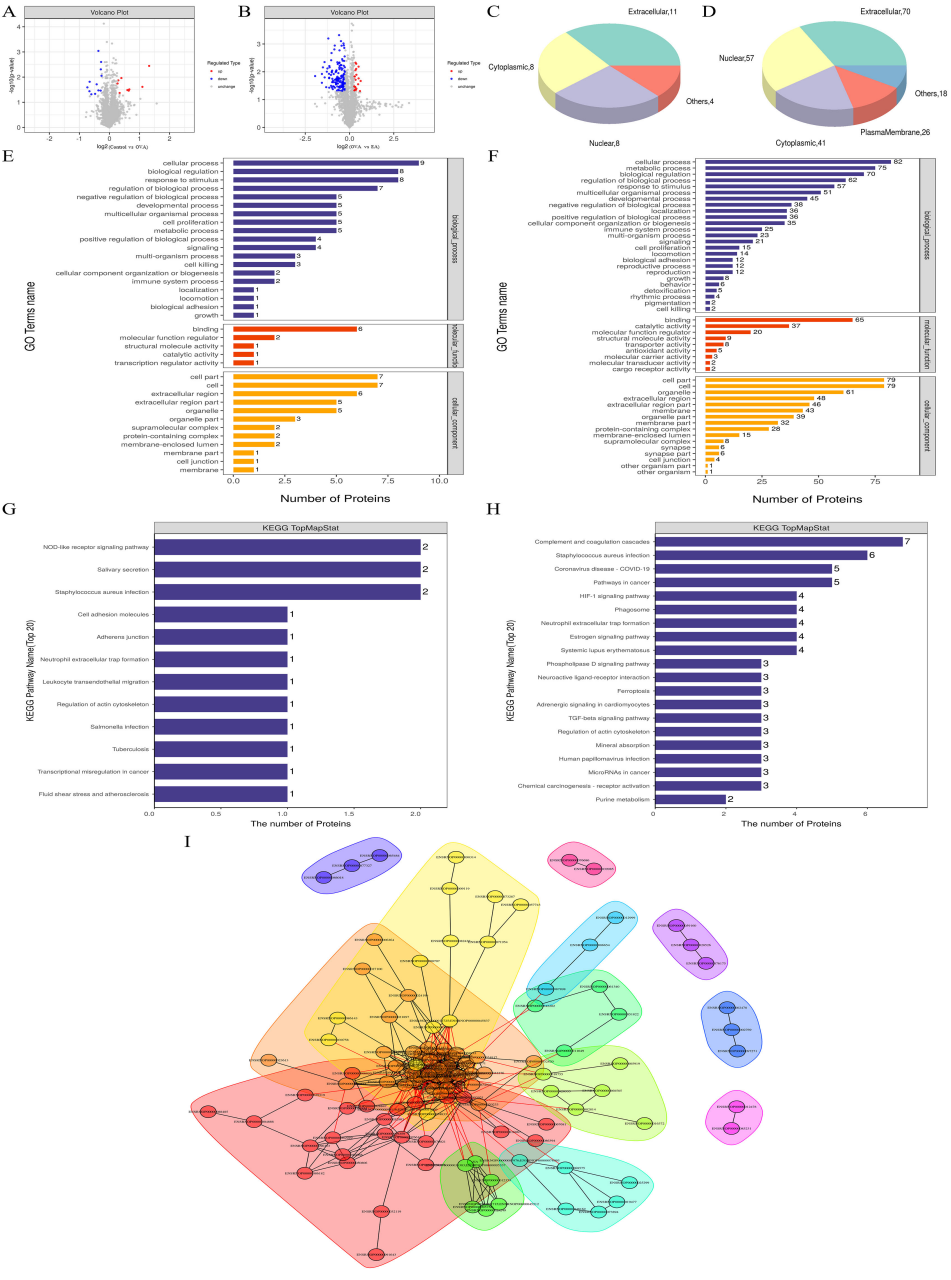
Results from proteomic analysis (n = 3 rats per group). **(A, B)** Volcano plots of Control vs. OVA **(A)** and OVA vs. EA **(B)**. Red dots show upregulated DEPs, blue dots show downregulated DEPs, and gray dots show proteins without substantial differential expression. **(C, D)** Subcellular localization pie charts of DEPs in Control vs. OVA **(C)** and OVA vs. EA **(D)**. **(E, F)** Gene Ontology (GO) annotation of DEPs in Control vs. OVA **(E)** and OVA vs. EA **(F)** by biological process (blue), molecular function (red), and cellular component (orange). **(G, H)** Kyoto Encyclopedia of Genes and Genomes (KEGG) pathway annotation statistics (top 20 pathways) of DEPs in Control vs. OVA **(G)** and OVA vs. EA **(H)**. **(I)** OVA vs. EA protein-protein interaction (PPI) network diagram of DEPs. Circled nodes are DEPs, and lines are protein–protein interactions. The circles show protein expression changes: blue for downregulated proteins, red for upregulated ones. The circles indicate network connectivity, with larger circles signifying enrichment analysis. DEPs, differentially expressed proteins; Control, control group; OVA, ovalbumin; EA, electroacupuncture.

A total of 3,333 proteins were identified in the quantitative proteomics analysis. P < 0.05 and fold change > 1.2 or < 0.83 served as the criteria to filter differential proteins across various groups. In the Control group relative to the OVA group, 9 proteins were upregulated, and 12 proteins were downregulated. In contrast, the EA group exhibited 24 upregulated and 151 downregulated differentially expressed proteins (DEPs) relative to that of the OVA group (**Figures 4A, B**; **Supplementary Figure 1**), among which the complement C3 precursor (pro-C3), procarboxypeptidase B2 (pro-CPB2), carboxypeptidase N subunit 1 (CPN1), and carboxypeptidase N subunit 2 (CPN2) were identified as significantly downregulated proteins. This result suggested their potential involvement in the alleviation of urticaria-like responses in the context of EA at LI11 and SP10. The subcellular localization prediction revealed that, in a comparison of the Control vs. OVA groups, the DEPs were primarily localized in the extracellular space (35.48%), cytoplasm (25.8%), and nucleus (25.8%). Similarly, DEPs in the EA vs. OVA comparison were found in the extracellular space (33.02%), nucleus (26.89%), and cytoplasm (19.34%) (**Figures 4C, D**).

### 3.3 Functional enrichment of DEPs

The 21 DEPs (Control vs. OVA) and 175 DEPs (EA vs. OVA) identified were subjected to functional enrichment analyses; the results revealed 132 and 1,395 significantly enriched GO terms in the DEPs, respectively (**Figures 4E, F**). These terms were clustered into biological processes, cellular components, and molecular functions. The KEGG enrichment analysis identified significantly enriched pathways between the Control and OVA groups that included saliva secretion and nucleotide-binding oligomerization domain (NOD)-like receptor signaling pathways. In contrast, a comparison between the OVA group and the EA group highlighted complement and coagulation cascades as well as neuroactive ligand-receptor interactions (**Figures 4G, H**). We then elucidated how these proteins interacted with one another and their roles in biological processes such as signal transduction, energy, and material metabolism using the STRING or IntAct database to analyze the protein–protein interaction (PPI) networks, with network visualization performed using Cytoscape software.

The PPI network of differential proteins between the OVA group and the EA group is presented in **Figure 4I**; **Supplementary Table 2**. We focused on the subset of highly connected nodes (degree count > 20), where the biological functions were primarily concentrated on participating in the dynamic balance of coagulation and the fibrinolysis system, the maintenance of the blood system and tissue repair homeostasis, material transportation, protease activity regulation, and support of immune and inflammatory responses. Examples included albumin, alpha-2-HS-glycoprotein, fibrinogen gamma chain, and alpha-1-microglobulin. Albumin is the predominant protein in plasma, and it exhibits a significant lipid-binding capacity that inhibits the extravasation of intravascular fluids. Furthermore, it may impede the advancement of associated diseases through its anti-inflammatory properties, lipogenesis suppression, and metabolic regulation (27–29). Alpha-2-HS-glycoprotein is a glycoprotein that is primarily secreted by the liver, and it functions as a multifaceted protective factor that regulates macrophage polarization and diabetic microvascular complications (30) and alleviates inflammation and fibrosis (31, 32). Additionally, it is linked to the emergence of several liver-related metabolic disorders, such as adipose tissue inflammation and dyslipidemia (33). The PPI network of the Control and OVA groups demonstrated a low clustering degree that hindered an analysis of the protein network modules.

### 3.4 Identification of differentially expressed metabolites

**Figure 5:**
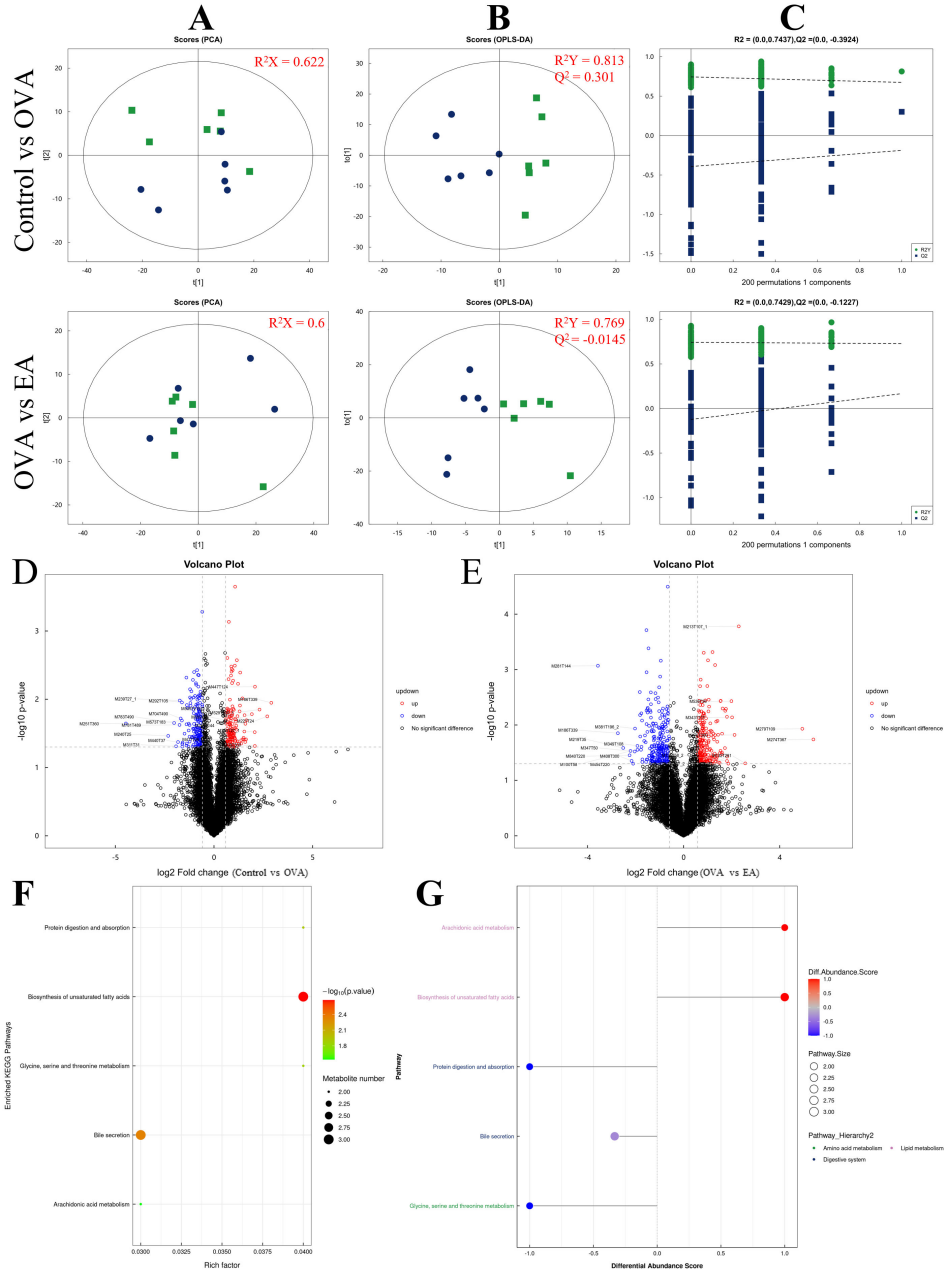
Results of metabolomics analysis (n = 6 rats per group). **(A)** Principal component analysis (PCA) score plot. t[1] and t[2] represent principal component 1 and principal component 2, respectively. The ellipses denote 95% confidence intervals. Points of the same color represent individual biological replicates within each group. **(B)** Orthogonal partial least squares discriminant analysis (OPLS-DA) score plot. t[1] and to[1] represent the predictive component and orthogonal component, respectively. The ellipses denote 95% confidence intervals. Points of the same color represent individual biological replicates within each group. **(C)** Permutation tests (n = 200) for OPLS-DA models. The x-axis indicates the permutation retention rate, and the y-axis represents R² and Q² values. Green dots denote R² (goodness of fit), while blue dots denote Q² (predictive ability). The dashed lines represent the regression lines. **(D, E)** Volcano plots comparing Control vs. OVA **(D, E)** and OVA vs. and EA **(F, G)** Kyoto Encyclopedia of Genes and Genomes (KEGG) enrichment analysis of differential metabolites between the OVA and EA groups **(F)** and their classification **(G)**. The color of the bubbles denotes the degree of enrichment across various metabolic pathways, whereas the size of the bubbles represents the number of enriched metabolites. Control, control group; OVA, ovalbumin; EA, electroacupuncture.

To investigate how EA affected UR rats’ serum metabolic profiles, LC-MS was used to detect metabolites in each group. A principal component analysis showed considerable group separation, as shown in **Figure 5A**. An orthogonal partial least squares discriminant analysis (OPLS-DA) was then performed to maximize the group discrimination and identify differential metabolites responsible for class separation. The OPLS-DA score plots revealed clear metabolic discrimination between groups (**Figure 5B**). This result indicated that the intervention coincided with alterations in the serum metabolic profiles of the UR rats. To validate the model robustness and prevent overfitting, 200 permutation tests were performed on the OPLS-DA model. The results showed that it did not exhibit overfitting (**Figure 5C**). The partial least squares discriminant analysis (PLS-DA) score plots and their corresponding 200 permutation tests are presented in **Supplementary Figure 2**.

Metabolites with differential expressions were identified using the integration of fold change and P values. Screening for the differential metabolites using the criteria of FC > 1.5 or FC < 0.67 and nominal P < 0.05 identified 320 metabolites that differed between the Control and OVA groups, as well as 447 metabolites that differed between the EA and OVA groups (**Figures 5D, E**). The 10 most significantly upregulated and downregulated differential metabolites were organized in descending order based on the log2 fold change. Their VIP, fold change, and P-values are presented in **Table 1**. The differentially expressed metabolites (DEMs) were primarily focused on lipids and lipid-like molecules, organic acids and derivatives, benzenoids, organic oxygen compounds, and organoheterocyclic compounds.

**Table 1:** Parameters of differential metabolites.

Superclass	Metabolites	VIP	FC	P	Group
Lipids and lipid-like molecules	Thymol-beta-d-glucoside	11.743	0.263	0.048	Control vs OVA
	Myristic acid	8.046	0.491	<0.01	
	2,3-dinor-8-isoprostaglandin-f2.alpha	3.088	1.340	0.014	
	Myristoleic acid	1.439	0.710	0.036	
	Pristanic acid	1.118	0.558	0.010	
	Hymeglusin	1.076	0.513	0.040	
	Cholic acid	7.292	0.368	0.036	OVA vs EA
	12s-hydroxy-5z,8z,10e,14z-eicosatetraenoic acid	6.322	2.312	0.046	
	Thromboxane b2	3.573	3.508	0.037	
	1-palmitoylglycerol	3.481	1.264	0.047	
	Eicosenoic acid	3.211	1.531	0.019	
	Nervonic acid	2.593	1.973	0.024	
	7.alpha.-hydroxy-3-oxo-4-cholestenoic acid	2.202	0.502	0.038	
	Oleoyl-l-carnitine	1.893	1.542	<0.01	
	Linoleoylcarnitine	1.574	1.765	<0.01	
	L-palmitoylcarnitine	1.558	1.524	0.015	
	Cis-13-docosenoic acid	1.477	1.502	0.040	
	Myristoleic acid	1.347	1.285	0.026	
	1-hexadecyl-2-(5z,8z,11z,14z-eicosatetraenoyl)-sn-glycero-3-phosphocholine	1.268	1.403	0.045	
	Pristanic acid	1.103	1.847	0.019	
Organic acids and derivatives	L-Lysine	10.792	0.536	0.032	Control vs OVA
	Betaine	5.491	0.739	0.047	OVA vs EA
	N-acetylglutamine	2.251	1.645	0.015	
	Ectoine	2.131	0.413	<0.01	
	Indoxyl sulfate	1.838	0.518	0.024	
Benzenoids	3-hydroxyanthranilic acid	1.702	0.585	0.042	Control vs OVA
	Salicylic acid	5.141	0.343	<0.01	OVA vs EA
	Siduron	3.922	1.610	0.034	
	Phenol	3.482	0.285	<0.01	
Organic oxygen compounds	Melibiose	1.299	0.588	0.014	Control vs OVA
Organoheterocyclic compounds	Adenine	1.349	0.580	0.035	OVA vs EA
Undefined	1-Hexadecanoyl-2-(9Z,12Z-octadecadienoyl)-sn-glycero-3-phosphoric acid	1.071	0.765	0.040	
Undefined	Cinchonine	2.161	0.627	0.032	Control vs OVA

VIP, Variable importance in the project; FC, Fold change; P, P-value; Control, control group; OVA, ovalbumin; EA, electroacupuncture.

### 3.5 KEGG functional enrichment and classification of differential metabolites

A pathway enrichment analysis was used to identify the relevant pathways based on the P value, enrichment count, and enrichment factor. A smaller P indicated a greater degree of enrichment. **Figures 5F, G** show the results of this analysis. In comparison to the OVA group, an analysis of the EA group showed enrichment in several pathways, as follows: the biosynthesis of unsaturated fatty acids, bile secretion, protein digestion and absorption, glycine, serine, and threonine metabolism, and arachidonic acid metabolism. No enrichment results were observed between the Control and OVA groups.

### 3.6 Integrated analysis of differential metabolites and proteins

**Figure 6:**
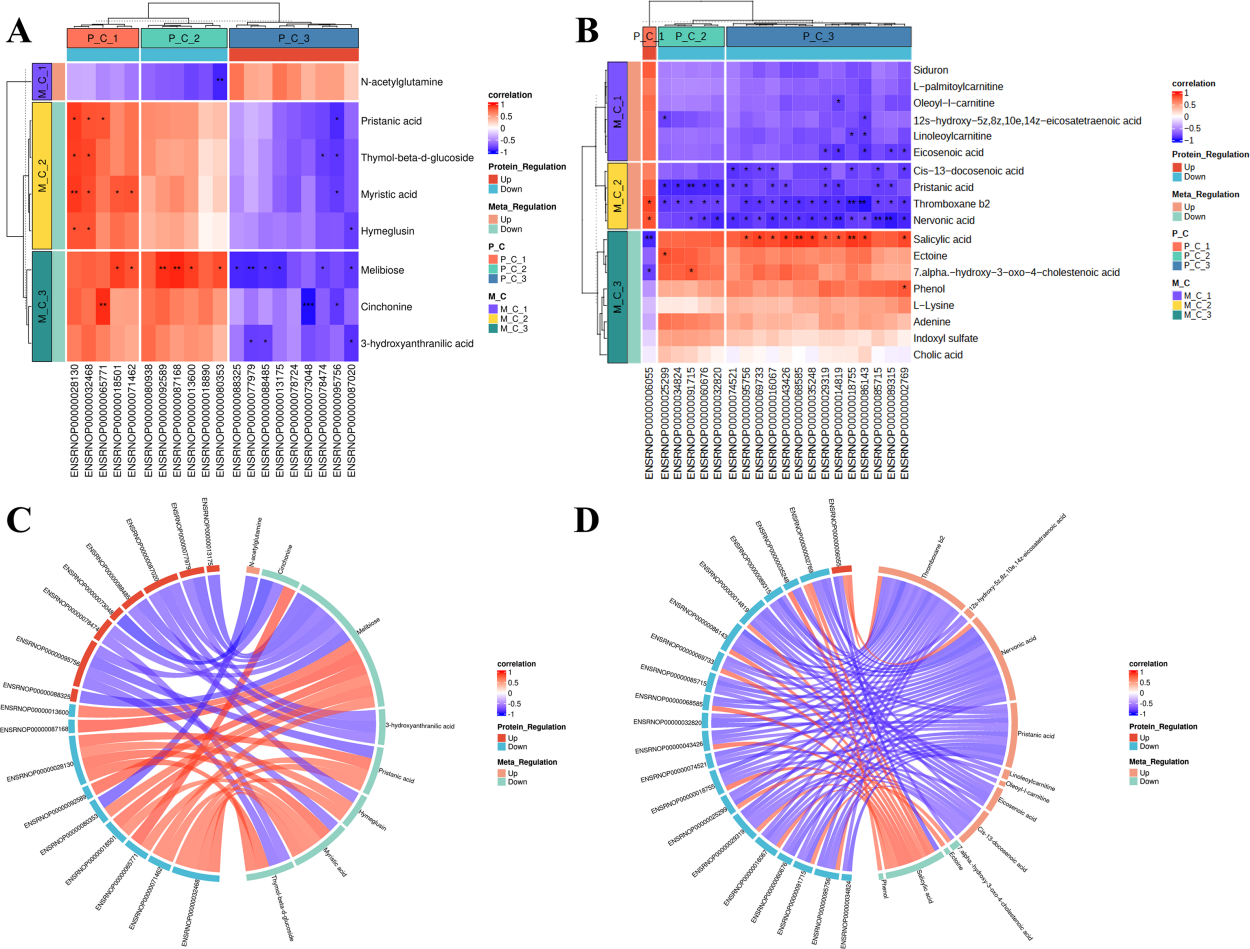
Correlation analysis of proteins and metabolites in matched sample sets from 9 rats (n = 3 per group). **(A, B)** Clustering heatmaps of the top 20 proteins and metabolites showing the relationships between Control and OVA **(A)** and OVA and EA **(B)**. Dark red indicates a strong positive association, whereas dark blue indicates a strong negative correlation. P_C, protein cluster; M_C, metabolite cluster. The asterisks indicate uncorrected P-values: *p < 0.05, **p < 0.01, ***p < 0.001. **(C, D)** Circos diagrams of proteins and metabolites (absolute correlation value > 0.8 and p-value < 0.05) depicting correlations between Control and OVA **(C)** and OVA and EA **(D)**. Red lines show positive relationships, and blue lines indicate negative correlations. Red sectors represent protein upregulation and metabolite higher abundance, and blue sectors represent protein downregulation and metabolite lower abundance. Control, control group; OVA, ovalbumin; EA, electroacupuncture.

We integrated correlation heatmap and chord diagram analyses to visualize the correlation patterns between the differentially expressed metabolites and proteins (**Figure 6**). Initially, the top 20 correlated proteins and serum metabolites were selected based on the Pearson’s correlation coefficients to construct the heatmap. The asterisks indicate uncorrected P-values: *p < 0.05, **p < 0.01, ***p < 0.001. The protein-metabolite pairs with absolute correlation coefficients greater than 0.8 and p-values less than 0.05 were filtered to generate the chord diagrams to show the association patterns in the Control vs. OVA groups (**Figures 6A, C**) and the OVA vs. EA groups (**Figures 6B, D**). A comparison between the Control and OVA groups (**Figures 6A, C**) showed that five upregulated proteins, including Serpina3m (ENSRNOP00000013175) and Cpm (ENSRNOP00000078474), showed positive correlations with N-acetylglutamine. Serpina3m (alpha-1-antichymotrypsin, ACT) is an endogenous protective protein that rapidly increases during acute inflammatory responses (34, 35) and prevents tissue damage by inhibiting chymotrypsin-like serine proteases (36, 37). Carboxypeptidase M (Cpm) inactivates complement anaphylatoxins C3a and C5a by cleaving their C-terminal arginine residues, and it suppresses excessive inflammatory reactions (38, 39). N-Acetylglutamine is an N-acetylated derivative of glutamine. During infection and/or hypercatabolic states, immune cells exhibit glutamine uptake rates equal to or greater than those of glucose (40, 41). Glutamine activates immune cells and promotes proliferation through ERK/JNK signaling pathway activation (42, 43) and provides glutamate for glutathione (GSH) synthesis (together with glycine and cysteine) to maintain redox homeostasis (44). A deficiency weakens the antioxidant protection of the glutamine-GSH axis, reduces reactive oxygen species (ROS) clearance, and affects inflammatory resolution and tissue repair. Conversely, the blue clusters in the heatmap and negative correlation lines in the chord diagram showed negative associations between the aforementioned upregulated proteins and lipid metabolism-related molecules (myristic acid, pristanic acid), tryptophan metabolism-related molecules (3-hydroxyanthranilic acid), and carbohydrate metabolism-related molecules (melibiose).

In a comparison between the OVA and EA groups (**Figures 6B, D**), fatty acid metabolism-related metabolites (nervonic acid, cis-13-docosenoic/erucic acid, eicosenoic acid, and pristanic acid) showed positive correlations with the Clec3b (ENSRNOP00000006055) protein and negative correlations with Klk6 (ENSRNOP00000060676) and Klk13 (ENSRNOP00000032820). Clec3b is a secreted glycoprotein that regulates fibrinolysis and tissue remodeling. It modulates extracellular matrix (ECM) remodeling and fibrosis through an interaction with collagen and promotes tissue repair and bone mineralization (45, 46). Klk6 and Klk13 belong to the kallikrein-related peptidase (KLK) family of secreted serine proteases, and they participate in tissue remodeling through ECM protein degradation (47, 48). Klk6 also regulates inflammatory responses through protease-activated receptor activation (49). Furthermore, the upregulated metabolite, thromboxane B2 (TXB2), showed a positive correlation with Clec3b and negative correlations with Klk6 and Klk13. Thromboxane B2 is the chemically stable, inactive metabolite of thromboxane A2 (TXA2). TXA2 is an eicosanoid lipid mediator generated from arachidonic acid through the sequential catalysis by phospholipase A2, cyclooxygenase COX-1/COX-2, and thromboxane synthase (TXAS) (50). TXA2 stimulates platelet activation and aggregation and functions as a vasoconstrictor during tissue injury and inflammation (50). It should be emphasized that these protein-metabolite correlations are strictly statistical and do not imply functional synergy, co-regulation, or causal directionality between the paired molecules.

### 3.7 Alterations in the C3a/C5a-C3aR/C5aR axis associated with EA at LI11 and SP10 in the OVA-induced acute urticaria-like model

**Figure 7:**
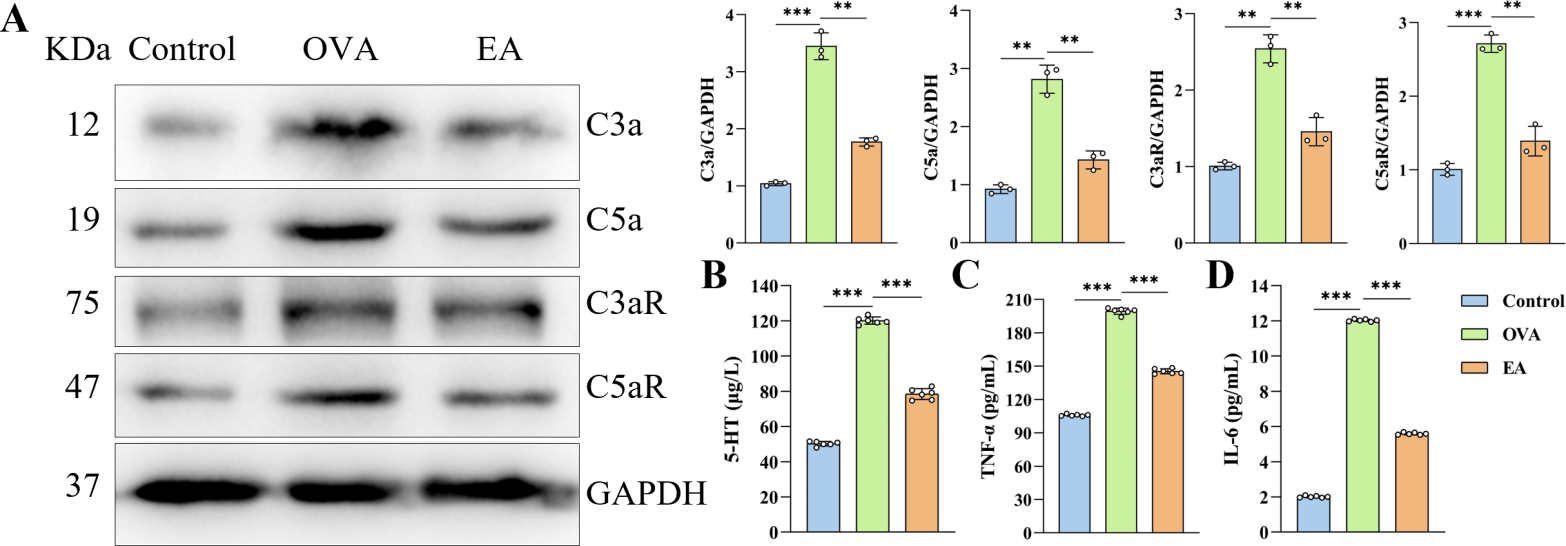
Alterations in the C3a/C5a-C3aR/C5aR axis in skin tissues and inflammatory mediators in serum coinciding with electroacupuncture in OVA-induced acute urticaria-like rats. **(A)** Representative blots and quantification of the protein expression levels of complement component 3a, complement component 5a, C3a receptor, and C5a receptor in skin tissues of the control, OVA, and EA groups (n = 3 rats per group; Western blot performed in technical triplicate). **(B–D)** Concentrations of 5-hydroxytryptamine **(B)**, tumor necrosis factor-α **(C)**, and interleukin-6 **(D)** in serum measured by enzyme-linked immunosorbent assay (μg/L for 5-HT; pg/mL for TNF-α and IL-6; n = 6 rats per group).. Quantifications are normalized to glyceraldehyde-3-phosphate dehydrogenase (GAPDH). Data are presented as means ± standard deviations, **p < 0.01, ***p < 0.0001. Control, control group; OVA, ovalbumin; EA, electroacupuncture.

The proteomic and metabolomic analysis results prompted us to examine the C3a, C5a, C3aR, and C5aR expression levels in the skin tissue using western blotting (**Figure 7A**). Compared to the OVA group, the C3a, C5a, C3aR, and C5aR protein expression levels were significantly reduced in the EA group (p < 0.01). This result indicated decreased expression of components of the C3a/C5a-C3aR/C5aR axis in the skin tissue of the EA group relative to the OVA group. An ELISA analysis of the serum indicated significant decreases in the levels of inflammatory mediators (TNF-α, IL-6, and 5-HT) in the EA group compared to the OVA group (**Figures 7B–D**, P < 0.0001). These results suggested that this intervention paralleled reduced expression of components of the C3a/C5a-C3aR/C5aR axis and lower levels of concomitant inflammatory mediators in the OVA-induced PCA acute urticaria-like model. This result was consistent with the omics data results.

## 4 Discussion

Acute urticaria manifests as pruritic wheals and/or angioedema that are typically triggered by IgE-mediated allergic reactions. Antihistamines are the standard of care for acute urticaria, but suboptimal responses and adverse reactions in some patients highlight the need to characterize molecular alterations linked to electroacupuncture in acute allergic models. In this study, we employed proteomic and metabolomic analyses to characterize molecular profiling alterations following an EA intervention at LI11 and SP10 in an acute IgE-mediated urticaria-like model and to identify acute-phase molecular indicators connected to symptom alleviation. The results showed that following the intradermal OVA injection, rats exhibited expanded wheal areas and increased scratching behaviors, with concomitant elevated histamine and IgE levels. These findings supported the successful establishment of the acute urticaria-like model. This intervention was associated with reduced wheal areas, decreased scratching frequency, and decreased histamine and IgE levels.

Mast cells are the primary effector cells in acute IgE-mediated reactions. They release preformed and rapidly synthesized inflammatory mediators upon degranulation. The toluidine blue staining revealed reduced mast cell degranulation rates following this intervention. Furthermore, the multi-omics analysis indicated that this treatment altered the lipid and amino acid metabolic profiles. This was accompanied by attenuated cutaneous pathological damage and decreased inflammatory indices.

While patients with chronic spontaneous urticaria frequently exhibit lipid metabolism disorders, particularly in unsaturated fatty acid biosynthesis and arachidonic acid metabolism (51, 52), the metabolic features of acute IgE-mediated hypersensitivity are mechanistically distinct and remain poorly understood. Therefore, in this study, we employed an OVA-induced PCA model to characterize the early metabolic alterations during acute urticaria-like reactions, with findings strictly confined to this acute experimental paradigm. Differential metabolites between the OVA and EA groups were predominantly enriched in lipid metabolism-related pathways that included unsaturated fatty acid biosynthesis, bile secretion, and arachidonic acid metabolism. These results implied that this intervention might correlate with lipid metabolic alterations in acute urticaria-like reactions. However, the underlying mechanism remains elusive. Compared with the OVA group, the unsaturated fatty acid biosynthesis pathway was upregulated following treatment, with erucic acid, eicosenoic acid, and nervonic acid exhibiting the most pronounced alterations. Within the context of acute inflammation, eicosenoic acid can be elongated to produce erucic acid (53), and this serves as a direct precursor for nervonic acid (54). These metabolites may participate in the synthesis of lipid mediators and modulate inflammatory and immune responses (55), thereby potentially influencing mast cell activation. However, this hypothesis requires experimental validation. The differential protein, Elovl4, is involved in the synthesis of docosahexaenoic acid (DHA) and eicosapentaenoic acid (EPA), and the reduced TNF-α levels observed in the EA group paralleled the reported anti-inflammatory effects of Elovl4 (56). The precise contribution of DHA and EPA to these effects requires further investigation.

Notably, intestinal flora disruption is a significant catalyst for aberrant lipid metabolism and the onset of chronic spontaneous urticaria (51, 52, 57), conditions characterized by chronic immune dysregulation and persistent metabolic disturbances distinct from the transient IgE-mediated reactions in the present PCA model. Previous studies have reported that while arachidonic acid may influence microbial metabolism in Bacteroides species (58, 59), the acute PCA model primarily reflected immediate-type IgE-mediated mast cell reactions. Whether the observed metabolic alterations are directly regulated by the intestinal microbiota remains to be determined. Moreover, C5a may influence inflammatory cascades through stimulation of the lipoxygenase pathway (60, 61). In the present study, metabolomic profiling additionally identified alterations in lipoxins and bile acid metabolism-related molecules (62–64); however, no functional relationship between these metabolic changes and the observed C5a alterations was established.

During acute IgE-mediated hypersensitivity reactions, mast cell activation commonly coincides with alterations in the amino acid metabolic microenvironment. In this study, omics analysis revealed that this intervention was associated with changes in protein digestion and absorption, glycine-serine-threonine metabolism, and protease activity. These metabolic alterations involved molecules such as betaine, L-lysine, and carboxypeptidase B2 (CPB2), which suggested that this intervention may co-occur with alterations in metabolic profiles and immune-related mediators in acute urticaria-like reactions. However, the exact mechanisms remain to be elucidated. Amino acid metabolism is closely linked to T cell and immune cell activation (65), and aberrant T cell activation is associated with chronic spontaneous urticaria (66). In this study, we focused on mast cell responses in the acute PCA model without the incorporation of a T cell subset analysis. Consequently, these observations should not be extrapolated to the pathological processes of adaptive immunity-mediated chronic urticaria. Betaine has been reported to alleviate oxidative stress through sulfur-containing amino acid metabolism modulation (67). Building upon our previous findings regarding the EA-mediated inhibition of NF-κB/NLRP3 (68), we hypothesized that alterations in betaine levels may have contributed to the anti-inflammatory effects observed in this study. However, the individual role of this pathway requires direct validation. L-lysine levels were altered following the treatment. Given that L-lysine antagonizes 5-HT binding to the 5-HT4 receptor (69), and 5-HT serves as a pro-inflammatory mediator that is released by mast cells (70), we hypothesized that this intervention may imply a link to modulation of 5-HT-mediated neurogenic or inflammatory signaling via the lysine-5-HT axis. However, this hypothesis requires experimental validation. Procarboxypeptidase B2 (proCPB2) undergoes enzymatic hydrolysis to generate active CPB2 that participates in complement and coagulation pathways. CPB2 exerts anti-inflammatory effects by inactivating C3a/C5a (71, 72), whereas C3a/C5a promotes pro-inflammatory responses via binding to C3aR/C5aR on mast cells (73, 74). The alterations in the CPB2 levels correlated with attenuated complement-driven mast cell activation in this model, but CPB2 additionally participates in bradykinin degradation and vascular barrier maintenance (71). These functions were not evaluated in this study.

Our data showed that, in the OVA-induced acute urticaria-like model, this therapy was accompanied by alterations in lipid and amino acid metabolic profiles. This co-occurred with alleviation of mast cell-mediated inflammatory responses. Previous studies have reported that arachidonic acid and lysine may synergistically promote the production of short-chain fatty acids by intestinal microbiota and inhibit both IgE- and non-IgE-mediated mast cell degranulation (75, 76). Betaine has been shown to suppress the PI3K/PDK1/AKT pathway, whereas erucic acid and nervonic acid may inhibit the p38 MAPK pathway, thereby contributing to the suppression of NF-κB activity. However, the activation status of these signaling pathways was not directly validated by the western blot or phosphorylation assays in this study. Given the well-established role of complement activation, particularly the C3a/C5a-C3aR/C5aR axis, in acute IgE-mediated hypersensitivity, we hypothesized that the observed electroacupuncture-associated changes might be linked to altered expression of components of this axis, though direct causal evidence remains to be established.

The complement system serves as a central effector mechanism of innate immunity and is closely associated with the pathogenesis of inflammatory diseases (77–79). In this study, the western blot and ELISA analyses showed decreases in C3a/C5a and C3aR/C5aR expression in skin tissues, as well as in serum concentrations of downstream inflammatory mediators (IL-6, TNF-α, and 5-HT), following EA at LI11 and SP10. C3a and C5a signal through their specific receptors, C3aR and C5aR, respectively, to promote increased vascular permeability and mast cell activation during acute allergic reactions (80). Notably, the proteomic analysis detected altered levels of the complement C3 precursor (pro-C3) following electroacupuncture (P < 0.05). Whether this reflected changes in the synthesis, degradation, or consumption remains to be determined. Hence, the relationship between pro-C3 alterations and the observed reduction in C3a/C5a warrants further mechanistic validation.

Several limitations of the present study warrant consideration. First, the OVA-induced PCA model reflected acute IgE-mediated hypersensitivity, and the results should not be extrapolated to chronic urticaria or other subtypes. Second, the absence of sham controls (e.g., non-acupoint stimulation or non-electrical needling) precludes definitive conclusions regarding acupoint specificity versus non-specific effects of restraint, needling, electrical stimulation, stress, or somatosensory activation. Accordingly, EA-related findings should be interpreted as intervention-package effects rather than actions specific to LI11 and SP10. Third, the complement-related findings represent exploratory correlative associations. Without functional validation (e.g., C3aR/C5aR antagonists, pathway inhibitors, or genetic approaches), these observations do not establish that changes in C3a/C5a and their receptors (C3aR/C5aR) mediate the therapeutic effects of EA, but rather provide candidate molecular indicators for hypothesis generation in future mechanistic studies. Fourth, limited biological replication and reliance on nominal P < 0.05 thresholds meant that no hits survived stringent FDR correction (FDR < 0.05) in the control vs. OVA comparison. These omics data are therefore presented strictly as hypothesis-generating exploratory screening, rather than as definitive evidence for a regulated mechanisms involving complement signaling and metabolic alterations.

## 5 Conclusion

In summary, in the OVA-induced acute urticaria-like model, this intervention was associated with the alleviation of inflammatory responses. The integrated proteomic and metabolomic analyses suggested that these observed changes may coincide with alterations in amino acid and lipid metabolic pathways. Decreased expression of components of the C3a/C5a-C3aR/C5aR axis in skin tissues along with reduced serum levels of downstream inflammatory mediators (IL-6, TNF-α, and 5-HT) represented exploratory biomarker associations coinciding with this therapeutic approach, rather than evidence of direct modulation of signaling via this axis or its downstream inflammatory networks. As this study was conducted using an acute PCA model, these findings are confined to this acute experimental paradigm and cannot be extrapolated to chronic urticaria or clinical therapeutic targets.

## Data Availability

The mass spectrometry proteomics data have been deposited to the iProX repository (https://www.iprox.cn/) with the project identifier IPX0016196000, which has been assigned the ProteomeXchange accession number PXD075803. The metabolomics data have been deposited to the MetaboLights database (https://www.ebi.ac.uk/metabolights/) with the dataset identifier MTBLS14327.
