# Supplementary figures and images for: Electroacupuncture at LI11 and SP10 is associated with alleviation of acute urticaria-like reactions in passive cutaneous anaphylaxis: an exploratory analysis of complement-related proteins and multiscale omics
Source: Front Immunol. 2026 Jun 8;17:1777806. doi: 10.3389/fimmu.2026.1777806 (PMC13284148; doi:10.3389/fimmu.2026.1777806)

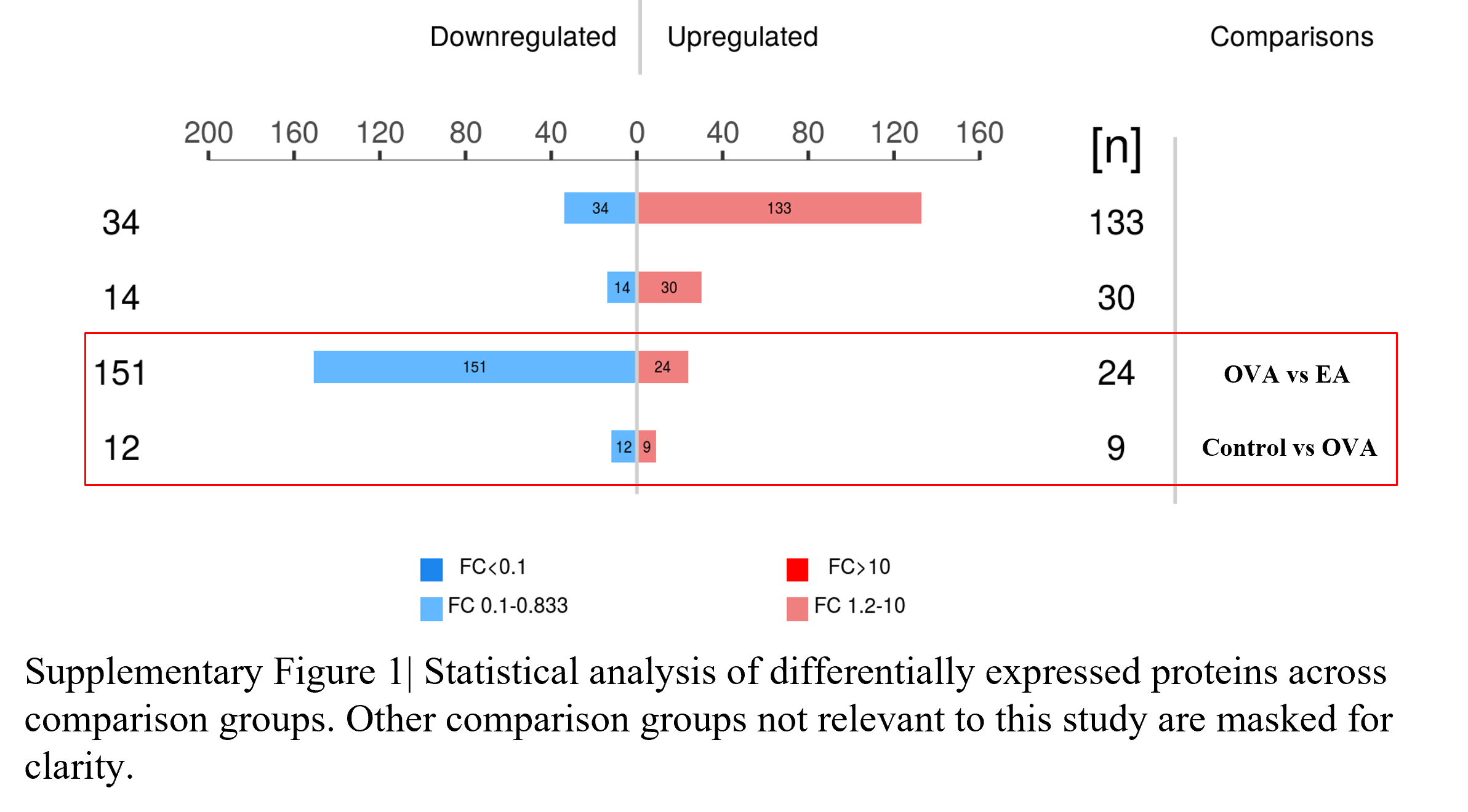

Supplement: Supplementary file 1 [file Image1.tif]

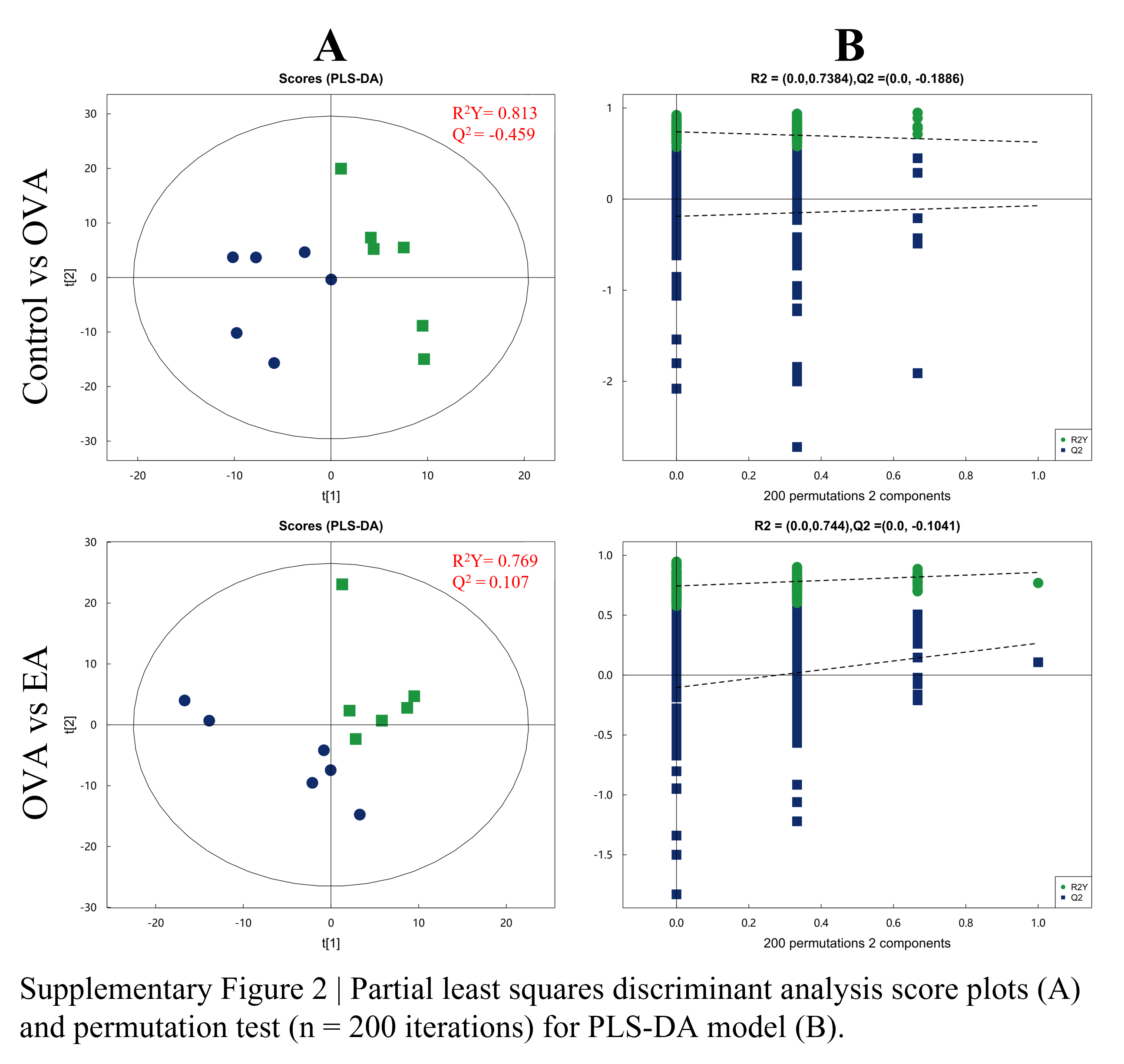

Supplement: Supplementary file 2 [file Image2.tif]
