# Supplementary material for: Electroacupuncture at LI11 and SP10 is associated with alleviation of acute urticaria-like reactions in passive cutaneous anaphylaxis: an exploratory analysis of complement-related proteins and multiscale omics
Source: Front Immunol. 2026 Jun 8;17:1777806. doi: 10.3389/fimmu.2026.1777806 (PMC13284148; doi:10.3389/fimmu.2026.1777806)
Supplement: Supplementary file 3 [file Table1.docx]

Supplementary Table 1 | Parameters of ion scanning mass spectrometry.

| **Parameters** | **Positiveion** | **Negativeion** |
| --- | --- | --- |
| Nebulizer Gas (GS1, PSI) | 60 | 60 |
| Auxiliary Gas (GS2, PSI) | 60 | 60 |
| Curtain Gas (CUR, PSI） | 30 | 30 |
| Ion Source Temperature (℃) | 600 | 600 |
| Ion Spray Voltage (V) | 5500 | -5500 |
| Mass Scan Range (TOF MS scan) | 80-1200 | 80-1200 |
| Mass Scan Range (Product Ion scan) | 70-1200 | 70-1200 |
