# Supplementary material for: Electroacupuncture at LI11 and SP10 is associated with alleviation of acute urticaria-like reactions in passive cutaneous anaphylaxis: an exploratory analysis of complement-related proteins and multiscale omics
Source: Front Immunol. 2026 Jun 8;17:1777806. doi: 10.3389/fimmu.2026.1777806 (PMC13284148; doi:10.3389/fimmu.2026.1777806)
Supplement: Supplementary file 4 [file Table2.docx]

Supplementary Table 2 | Protein protein interactions (PPI) of differentially expressed proteins in plasma between OVA group and electroacupuncture group.

| **Protein** | **Degree** | **Protein Name** | **Gene Name** |
| --- | --- | --- | --- |
| ENSRNOP00000003921 | 39 | albumin | Alb |
| ENSRNOP00000055223 | 34 | alpha-2-HS-glycoprotein | AABR07034632.1 |
| ENSRNOP00000083556 | 31 | plasminogen | Plg |
| ENSRNOP00000082495 | 30 | fibrinogen gamma chain | Fgg |
| ENSRNOP00000009248 | 29 | alpha-1-microglobulin/bikunin precursor | Ambp |
| ENSRNOP00000050663 | 27 | fibrinogen beta chain | Fgb |
| ENSRNOP00000068933 | 27 | apolipoprotein A1 | Apoa1 |
| ENSRNOP00000066934 | 27 | complement C3 | C3 |
| ENSRNOP00000060007 | 26 | fibrinogen alpha chain | Fga |
| ENSRNOP00000014909 | 25 | carboxypeptidase B2 | Cpb2 |
| ENSRNOP00000089330 | 25 | ceruloplasmin | Cp |
| ENSRNOP00000082662 | 24 | inter-alpha-trypsin inhibitor heavychain 2 | Itih2 |
| ENSRNOP00000022113 | 21 | transthyretin | Ttr |
| ENSRNOP00000071702 | 21 | apolipoprotein E | Apoe |
| ENSRNOP00000012092 | 20 | amyloid P component, serum | Apcs |
| ENSRNOP00000013896 | 20 | serine (or cysteine) proteinase inhibitor, clade A, member 3C | Serpina3c |
| ENSRNOP00000070308 | 20 | Serine protease inhibitor | Serpina3n |
